# Identifying spatiotemporal hotspots and sources of small-middle riverine nitrogen pollution using intensive field sampling and export coefficient model

**DOI:** 10.7717/peerj.21512

**Published:** 2026-07-29

**Authors:** Zipeng Zhang, Rongyue Ma, Guangyu Su, Yuxin Fan, Simiao Wang, Jiawen Sun, Tianxiang Wang

**Affiliations:** 1 National Marine Environmental Monitoring Center, Dalian, China; 2 School of Chemical Engineering, Ocean and Life Sciences, Dalian University of Technology, Panjin, China

**Keywords:** Spatiotemporal hotspots, Intensive field sampling, Export coefficient model, Riverine nitrogen pollution

## Abstract

Terrestrial pollution discharge induces spatiotemporal heterogeneity in riverine nitrogen dynamics. Accurately identifying sensitive temporal phases, spatial hotspots, and primary pollution sources is essential for effective riverine environmental management. This study developed an identification framework to delineate spatiotemporal pollution hotspots and source contributions in the Dai River basin by coupling intensive monitoring with the export coefficient method. The results indicated that the flood season was a critical period for increased total nitrogen (TN) pollution. Approximately 60% of the annual rainfall occurred in July and August, resulting in a 2.5-fold increase in TN concentrations in August compared to June. Moreover, rainfall events exceeding 100 mm/day led to 2- to 3.5-fold increases in TN concentrations relative to pre-event levels, with maximum increases surpassing 14-fold. Automatic monitoring data revealed a decreasing trend in TN concentrations from upstream to downstream. Intensive monitoring identified the Yu, Xidai, and Sha rivers at the headwaters as pollution hotspot zones, characterized by higher nitrogen concentrations and larger basin areas. The export coefficient model results showed that urban domestic wastewater and livestock farming were the dominant sources, accounting for 35.87% and 35.55% of total discharges, respectively. Yuguan town exported 33.34 t of nitrogen, contributing 47% of the total discharges. Based on hotspot identification, we proposed differentiated and integrated management measures for each town.

## Introduction

Nitrogen pollution is one of the major challenges affecting river systems, excessive nitrogen inputs can lead to eutrophication and the deterioration of water quality (Wang, Zhang & He, 2024). Riverine nitrogen pollution originates from a wide range of sources, including urban and rural domestic wastewater, agriculture, livestock farming, and other related activities (Wang, Zhang & He, 2024). These sources exhibit distinct discharge patterns and mechanisms, resulting in complex and variable environmental impacts. Municipal and industrial wastewater are discharged in the form of point sources through fixed sewer outlets, influenced by factors such as population size, production scale, production efficiency, and wastewater treatment capacity (Matiatos et al., 2023). Other sources are discharged as nonpoint sources and are dispersed throughout the watershed, exhibiting significant spatial and temporal heterogeneity and being driven by rainfall (Li et al., 2023a; Li et al., 2023b). Riverine nitrogen pollution is also affected by hydrological connectivity. Nitrogen discharged upstream can be transported downstream *via* runoff (Kim et al., 2023). The complex pollution sources and discharge processes exacerbate the sensitivity of riverine nitrogen pollution changes. Identifying spatial and temporal hotspots and major sources of nitrogen pollution is crucial for effective riverine environmental management.

Water quality monitoring is a fundamental regulatory approach for detecting river pollution (Bieroza et al., 2023). Currently, most monitoring efforts are conducted at river outlets, which prevents accurate identification of specific river sections and pollution sources (Chellaiah et al., 2024; Li et al., 2023a; Li et al., 2023b). The Dai river is an important coastal river in Qinhuangdao city that flows directly into the sea. In recent years, total nitrogen pollution in the river has become severe, with total nitrogen (TN) concentrations at the estuary consistently exceeding regulatory limits. Since the water quality of the Dai river directly affects the nearshore ecosystem, controlling TN pollution has become a priority for regional water environment management. The Soil and Water Assessment Tool (SWAT) model integrates topography, land use, and rainfall to simulate hydrological and sediment transport processes, enabling rapid estimation of pollution outputs from hydrologic response units (Keller et al., 2023). Zhang et al. (2022) employed the SWAT model to analyze the sources of TN pollution in Luoyang city, identifying atmospheric deposition, fertilizer application, and rural sewage as the primary contributors. Zhang et al. (2024a) and Zhang et al. (2024b) used the SWAT model to evaluate variations in nitrogen pollution sources in Jincheng City under different land use/land cover scenarios and found that nitrogen outputs from fertilizer use increased from 29.8% in 1997 to 35.6% in 2022. Storm Water Management Model (SWMM) and MIKE URBAN offer advantages in simulating the migration processes and pollutant fluxes of domestic wastewater by incorporating urban drainage networks (Alamdari et al., 2022; Zhang et al., 2023a; Zhang et al., 2023b; Zheng et al., 2024). Guo et al. (2024) employed the SWMM model to investigate pollutant contributions from impervious surfaces, pipelines, and channels during rainfall events in Wuxi. Pipelines were found to be a significant source of pollution, accounting for 59% of TN, 83% of ammonium nitrogen (NH_4_^+^–N), 90% of nitrate nitrogen (NO_3^−^_–N), and 77% of phosphate (PO_4_^3^−^^–P) in the total discharge. Peng et al. (2023) analyzed nonpoint source pollution in the Danjiang river using the MIKE model and found that TN was the dominant pollutant. However, it is necessary to note that these distributed models require extensive input data, which limits the practical application of the models (Keller et al., 2023). This obstacle is particularly pronounced for small and medium-sized rivers, where water quality and flow data are rarely monitored, introducing significant challenges for model validation. Urban areas similarly face limitations in data availability. Inconsistencies in construction and planning between newly developed and older urban zones increase the complexity of underground stormwater and sewer systems, hinder accurate characterization of pipe networks, and constrain the application of distributed urban water quality models (Wang et al., 2022; Ning et al., 2025). Remote sensing can rapidly estimate pollution export by analyzing unit environmental characteristics based on actual land use data (Huang et al., 2022). He et al. (2024) employed Sentinel-2 satellite imagery to assess the spatial distribution of chlorophyll-a, total nitrogen, total phosphorus, ammonia nitrogen, and chemical oxygen demand in Dong Lake. Zhang et al. (2024a), Zhang et al. (2024b) combined remote sensing with the SWAT model to analyze TN pollution in the sixth drainage ditch of the Yellow river irrigation area in Ningxia, found that agricultural irrigation contributed 92.88% of the pollution output. Currently, remote sensing interpretation methods are influenced by image accuracy and are suitable for areas where hydrological information is unavailable (Cao et al., 2023). The export coefficient model estimates pollutant loads from different land use types using predefined export coefficients (Zhang et al., 2023a; Zhang et al., 2023b). As an aggregate model, it is widely applied due to its relatively low data requirements (Rao et al., 2022). Wang et al. (2024a), Wang et al. (2024b) applied the export coefficient model to assess nonpoint source nitrogen discharges in the Jialing River Basin, which indicated an increase from 128,484 t in 2005 to 202,197 t in 2019. Zhang et al. (2023a) and Zhang et al. (2023b) assessed nitrogen discharges in the Yongji irrigation district using the export coefficient model and found that nitrogen from farmland accounted for 49% of the annual TN load. Calculation methods vary across different types of pollution sources (Pintos Andreoli et al., 2023). Domestic sewage calculations should account for pollutant reduction resulting from wastewater treatment. Agricultural non-point source discharges require consideration of various cultivation types. Industrial effluent discharge depends on both the volume of effluent and the concentration of pollutants.

Existing methods have explored river pollution identification from temporal, spatial, and source-based perspectives. However, small and medium-sized river basins often suffer from data scarcity, posing substantial challenges to the application of distributed models. Moreover, the sparse distribution of monitoring stations limits the ability to analyze the spatial characteristics of pollution. Currently, effective approaches for identifying pollution in these rivers remain inadequate. In recent years, relatively high frequency monitoring has been implemented in some large river basins. For example, Sheng et al. (2025) used automatic monitoring at 4 h intervals in the Pearl river basin and found that sampling frequency significantly affected TN source apportionment at 65.85% of the stations. Tian et al. (2025) employed near daily monitoring in the Yellow river basin and reported that about 70% of daily TN concentrations exceeded the Class V standard, with extreme rainfall events contributing 45–85% of the annual TN load. Other large basin studies (Wang et al., 2023a; Gao & Duan, 2026) have also relied on short term sampling. These studies remain focused on large river basins such as the Yangtze, Pearl, and Yellow rivers. For small and medium sized rivers, where monitoring infrastructure is weak, similar high resolution intensive monitoring is largely absent, making it difficult to identify pollution hotspots at the town scale or at confluences of mainstem and tributaries. Therefore, existing approaches are insufficient to support fine scale nitrogen pollution source apportionment and management in small and medium sized rivers. To address this gap, this article employs the Dai river as a case study to develop a pollution identification framework that combines intensive monitoring with the export coefficient method. The objectives are to identify spatiotemporal pollution hotspots, determine dominant nitrogen sources, and propose targeted control strategies to support water quality management in small and medium-sized rivers.

## Materials & Methods

### Study area

The Dai river, located in Qinhuangdao City, has a total length of 39 km and a watershed area of 282 km^2^. The average annual runoff is approximately 4.78 × 10^8^ m^3^, primarily sourced from rainwater, ice melt, and other natural waters. The river flows through six towns, including Yuguan, Tengfei street, Chuanchanglu street, Daihe, Niutouya, and Haibin, before ultimately discharging into the Bohai Sea. The average annual precipitation is 602.3 mm, with an uneven distribution concentrated between June and September, which accounts for 75% to 85% of the yearly total. Nitrogen pollution is the main environmental concern in the basin.

### Total nitrogen pollution identification framework for small and medium-sized rivers

**Figure 1:**
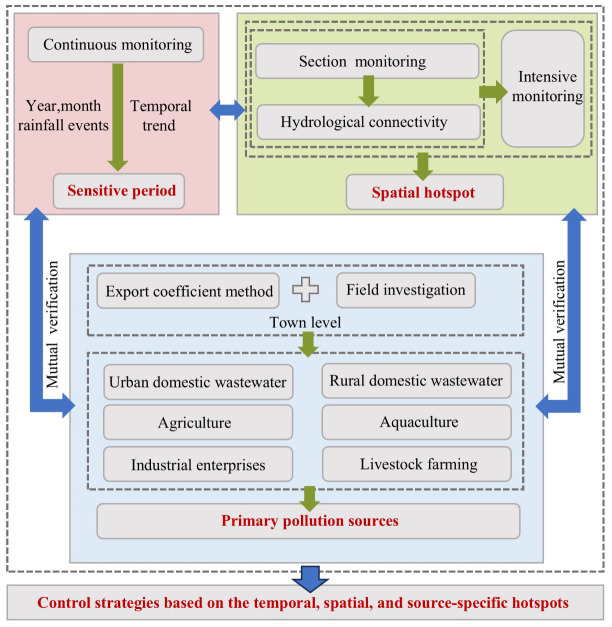
An integrated framework for identifying temporal, spatial, and source specific nitrogen pollution hotspots in small and medium sized rivers.

This study proposed a framework for identifying hotspots of TN pollution in small and medium-sized rivers (Fig. 1). First, temporal trends of TN pollution were analyzed using data from continuous monitoring stations, and rainfall information was used to identify sensitive periods of water quality variation. Second, spatial variations in riverine TN and runoff-driven transport processes were qualitatively assessed based on data from monitoring sections. Third, intensive field sampling was conducted to perform a detailed analysis of polluted reaches and to identify pollution hotspots. Fourth, pollutant loads from different sources were estimated at the town level using the export coefficient method to determine primary pollution sources and affected areas, which were then verified through field investigations and monitored water quality data. Finally, control strategies for riverine nitrogen pollution were proposed based on the identified temporal, spatial, and source-specific hotspots. In summary, we combined intensive monitoring, routine monitoring, and the export coefficient method to build a framework for identifying nitrogen pollution hotspots and sources at the town scale and at key mainstream tributary nodes in small and medium sized river basins.

### Water quality monitoring and intensive field sampling

**Figure 2:**
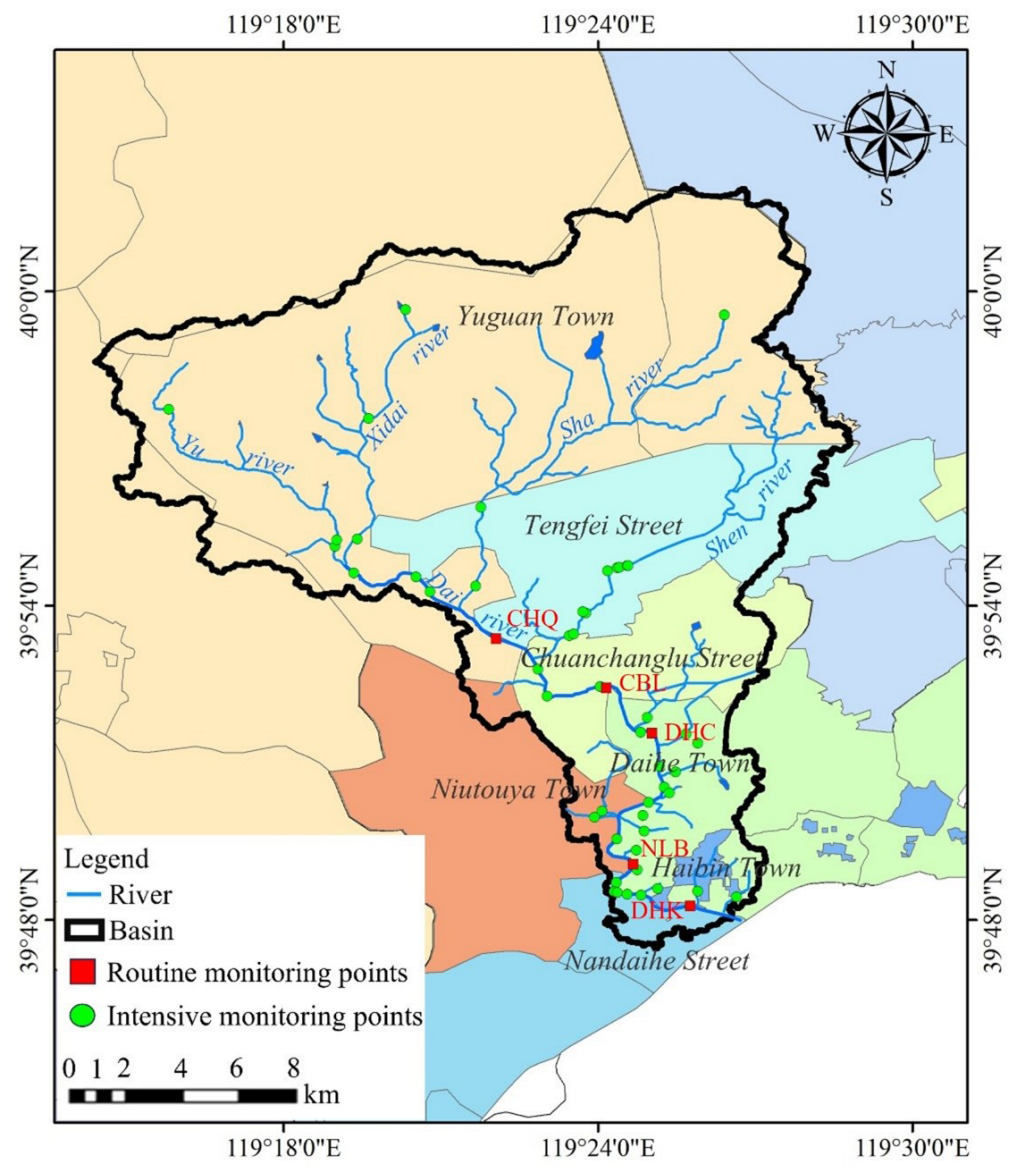
Spatial distribution of routine monitoring sections and intensive sampling points in the Dai river basin. Five routine monitoring sections are located along the mainstream. Fifty one intensive sampling points cover tributaries, river sources, confluences, and areas with environmental concerns.

Routine water quality monitoring in the Dai river basin was conducted from upstream to downstream through five monitoring sections: ShaHeQiao (SHQ), ChangBuLao (CBL), DaiHeChun (DHC), NiLongBa (NLB), and DaiHeKou (DHK) (Fig. 2), the data were sourced from the Qinhuangdao Ecological Environment Bureau. These sections focused on tracking pollutant transport processes within the mainstream. However, the existing monitoring scheme faced challenges in accurately assessing the influence of tributaries, making it difficult to identify pollution hotspots. To address this issue, we employed an intensive monitoring program to comprehensively observe water quality in both the mainstem and tributaries of the Dai river basin (Fig. 2). The field sampling for this study was conducted in publicly accessible water bodies and did not involve protected areas or private property.

The design principles for monitoring point placement were as follows: (1) at least one monitoring point was established for each tributary; (2) for rivers longer than 10 km, a monitoring point was set at the river source; (3) monitoring points were placed at the confluences where tributaries join the main river; (4) based on field investigations, monitoring points were located at sites with identified environmental concerns; and (5) if a significant change in pollutant concentration was observed between two monitoring points, additional points were added in the intermediate section. Based on these principles, a total of 51 monitoring points were sampled. The intensive field sampling was conducted in November 2022. Routine monitoring were sampled monthly. Water samples were collected using plexiglass samplers, immediately stored at 4 °C, and transported to the laboratory for analysis within 24 h. TN was determined using the alkaline potassium persulfate digestion method (HJ 636–2012). Following this method, water samples were digested at 120 °C for 30 min to convert nitrogenous compounds to nitrate. The absorbance was then measured at 220 nm and 275 nm, and the corrected absorbance (A220−A275) was used to calculate TN concentration based on a calibration curve. To evaluate analytical precision, each water sample was measured in triplicate, three subsamples were processed independently through the same procedure, and absorbance readings were recorded within 3 min of digestion completion. The relative standard deviation (RSD) of the corrected absorbance was calculated for each triplicate set, with an acceptance criterion of RSD ≤ 5% following standard quality assurance practices. The mean TN concentration of the three subsamples was reported for each sample.

### Export coefficient model

This article employed the export coefficient method (ECM) to calculate nitrogen pollution from various sources, including urban domestic sources, rural domestic sources, livestock farming, agriculture, aquaculture, and nitrogen-related industrial enterprises (Mao et al., 2023; Xia et al., 2025; Wang et al., 2024a; Wang et al., 2024b). The total pollution discharges were the sum of discharges from these sources. The ECM is a semi-empirical approach widely used to estimate nutrient loads from diffuse and point sources in a watershed. Its fundamental principle is that the total annual pollutant load delivered to a water body is the sum of the loads exported from each nutrient source within the catchment. The general form of the ECM is expressed as (Johnes, 1996):

\begin{eqnarray*}N=\sum _{i=1}^{n}{K}_{i}\times {A}_{i} \end{eqnarray*}

where *N* is the total annual pollutant load (t a^−1^), *K*_*i*_ is the export coefficient for the *i*th pollution source (*e.g.*, kg ha^−1^ a^−1^ for land use, kg head^−1^ a^−1^ for livestock, or g person^−1^ d^−1^ for domestic sewage), *A*_*i*_ is the activity level of the *i*th source (*e.g.*, area in ha, number of livestock, or population size), and *n* is the number of source types. Based on this principle, the following subsections detail the specific calculation methods for each source category in the Dai River basin. Meanwhile, to evaluate the uncertainty of the export coefficient model outputs, we performed a Monte Carlo simulation following the method of Wang et al. (2026).

### Urban and rural domestic sewage

Domestic sewage is discharged into the river in two pathways. One involves tailwater released after treatment, and the other involves the direct release of untreated wastewater. The relevant data were obtained from the Qinhuangdao Statistical Yearbook and field surveys.

\begin{eqnarray*}{N}_{WWTPs}& =PO{P}_{urban}\times {Q}_{urban}\times kQ\times 365\times kC\times {C}_{t}\times 1{0}^{-9} \end{eqnarray*}

\begin{eqnarray*}{N}_{\mathrm{urban}-\mathrm{untreated}}& =PO{P}_{urban}\times {Q}_{urban}\times kQ\times 365\times \left( 1-kC \right) \times {C}_{w}\times 1{0}^{-9} \end{eqnarray*}

where *N*_*WWTPs*_ is TN export from treated urban domestic wastewater (t), *N*_urban−untreated_ is TN export from untreated urban domestic wastewater (t), *POP*_*urban*_ is the urban population size, from the Qinhuangdao Statistical Yearbook, *Q*_*urban*_ is the urban per capita domestic water consumption the data are sourced from the Ministry of Ecology and Environment of the People’s Republic of China (2021), with a value of 145 L/day, *kQ* is the sewage discharge coefficient, the data are sourced from the Ministry of Ecology and Environment of the People’s Republic of China (2021a), with a value of 0.8, *kC* is the wastewater collection rate, *C*_*t*_ is the nitrogen concentration of tailwater (mg/L) derived from the Qinhuangdao wastewater treatment plant. and *C*_*w*_ is the nitrogen concentration of domestic wastewater (mg/L), with a value of 73.8 (mg/L).

\begin{eqnarray*}{N}_{\mathrm{rural}-\mathrm{treated}}& =PO{P}_{rural}\times {K}_{\mathrm{ruralpop}-\mathrm{N}}\times \left( 1-kR \right) \times 365\times 1{0}^{-6} \end{eqnarray*}

\begin{eqnarray*}{N}_{\mathrm{rural}-\mathrm{untreated}}& =PO{P}_{rural}\times {K}_{\mathrm{ruralpop}-\mathrm{N}}\times kR\times 365\times 1{0}^{-6} \end{eqnarray*}

where *N*_rural−treated_ is TN export from treated rural domestic wastewater (t), *N*_rural−untreated_ is TN export from untreated rural domestic wastewater (t), *POP*_*rural*_ is the rural population size; *K*_*ruralpop*−*N*_ is the rural domestic pollution production intensity (g/person day); and *kR* is the nitrogen pollution removal rate. The data of *K*_*ruralpop*−*N*_ and *kR* are sourced from the Ministry of Ecology and Environment of the People’s Republic of China (2021a) respectively taking values of 0.65 (g/person day) and 47%.

### Livestock

Nitrogen pollution from livestock farming (*N*_*livestock*_) is estimated based on the scale of breeding operations and pollutant export coefficients. The total nitrogen export coefficients for the four types of livestock under different farming scales are presented in Table 1. This paper considered four types of livestock and poultry, including cow, cattle, pigs, and chicken. The quantities of these livestock and poultry were sourced from Qinhuangdao Statistical Yearbook and field surveys.

**Table 1:** Total nitrogen export coefficients for livestock farming.

Livestock and poultry species	Total nitrogen	
	Large-scale farming	Individual farming
Cow (kg N individual-1)	11.4005	11.0903
Cattle (kg N individual-1)	5.3333	4.6367
Pig (kg N individual-1)	0.5186	0.1864
Chicken (kg N individual-1)	0.0630	0.0216
Data source	The coefficients were determined in accordance with the Manual of Production and Discharge Coefficients for Agricultural Pollution Sources (https://www.mee.gov.cn/xxgk2018/xxgk/xxgk01/202106/t20210618_839512.html).	

### Agriculture

TN loss from agriculture is estimated using the production and export coefficient method. It is calculated by multiplying the total sown area of crops and orchards by their corresponding pollutant export coefficients. The formula for calculating agricultural TN loss is as follows.

\begin{eqnarray*}{\mathrm{N}}_{agriculture}=\sum _{i=1}^{n}{A}_{i}\times {K}_{agr-i}\times 1{0}^{-3} \end{eqnarray*}

where *N*_*agriculture*_ is agricultural nitrogen pollution discharge (t), *A*_*i*_ is the area of the *i* crop (ha), and *K*_*agr*−*i*_ is the nitrogen export coefficient of the *i* crop (kg/ha), The TN export coefficient for cultivated land is 0.775 kg/ha, while that for garden land is 1.448 kg/ha, taken from the Ministry of Ecology and Environment of the People’s Republic of China (2021b).

### Aquaculture

The pollutant export from aquaculture refers to the amount of pollutants discharged directly into external water bodies, such as lakes, rivers, and oceans. The pollutant discharge coefficient is determined based on the values specified in the Ministry of Ecology and Environment of the People’s Republic of China (2021c), with the value of 2.588 kg/t. The aquaculture production was calculated based on relevant data from the statistical yearbook.

\begin{eqnarray*}{N}_{\mathrm{aquaculture}}={Q}_{aqu}\times {K}_{aqu}\times 1{0}^{3} \end{eqnarray*}

where *N*_aquaculture_ is aquacultural nitrogen pollution discharge (t), *Q*_*aqu*_ is the quantity of aquaculture (t) and *K*_*aqu*_ is the discharge coefficient for aquaculture (kg/t).

### Industrial wastewater

The nitrogen discharges from industrial enterprises are equal to the amount of wastewater discharged multiplied by the nitrogen concentration in the wastewater.

\begin{eqnarray*}{\mathrm{N}}_{\mathrm{enterprise}}=\sum _{i=1}^{n}{I}_{i}\times {C}_{i} \end{eqnarray*}

where *N*_*enterprise*_ is the industrial enterprise’s nitrogen discharges (t), *I*_*i*_ is the amount of wastewater discharged by the *i* enterprise (10^6^ t), and *C*_*i*_ is the concentration of wastewater, discharged by the *i* enterprise (mg/L).

## Results

### Temporal dynamics of riverine nitrogen pollution and the influence of the rainfall

**Figure 3:**
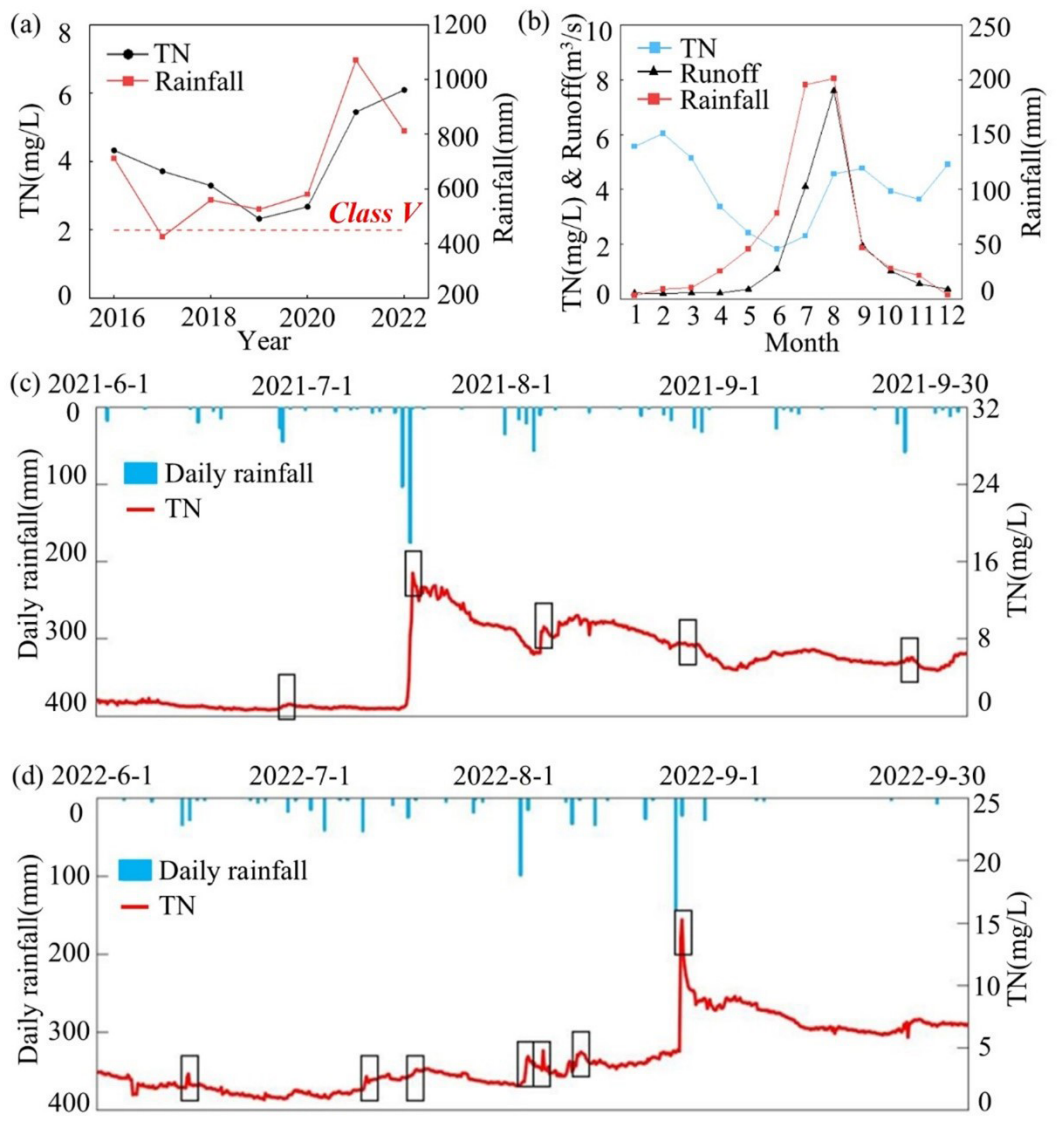
Annual, monthly, and daily trends of total nitrogen (TN) pollution and hydrological variations in the Dai river basin. (A) Annual TN concentrations and total annual rainfall from 2016 to 2022. The horizontal dashed line indicates the Class V water quality standard for TN (two mg/L, GB3838 2002). (B) Multi year average (2016–2022) monthly TN concentration, river runoff, and rainfall. (C) Daily rainfall and TN concentration at the Daihekou automatic monitoring station from 2021 to 2022. Rainfall events exceeding 100 mm are highlighted in red. (D) Scatter plot of TN concentration *versus* daily rainfall amount for 41 rainfall events. The dashed line shows a fitted power law relationship.

TN pollution in the Dai river section remains at a high level, with multi-year average concentrations exceeding twice the Class V water quality standard (GB3838-2002) (Fig. 3A). The interannual variation showed a gradual decrease from 2016 to 2019, followed by an increase, which may be linked to rainfall. Rainfall in the Daihe river basin decreased by 40% from 2016 to 2017, leading to a 48% reduction in runoff. This reduction weakened the scouring of non-point source pollution, thereby contributing to improved water quality. Along these lines, water quality would be expected to improve with the increased rainfall and runoff observed in 2018 and 2019 compared to 2017. However, the relatively low rainfall in 2017 resulted in dry antecedent soils, which enhanced rainfall retention in the soil. Consequently, both runoff generation and pollutant scouring capacities were reduced, contributing to a continued decline in TN concentrations in the river. In 2020, both rainfall and runoff continued to decline, yet TN concentrations increased—likely due to enhanced nutrient release from sediments under low-flow conditions. In 2021, rainfall increased significantly, leading to substantial rises in both runoff and TN concentrations. Although rainfall decreased in 2022 compared to 2021, TN concentrations continued to rise. This trend may be attributed to differences in the temporal distribution of rainfall. In 2021, rainfall occurred in two peaks—in mid-June and early September, while rainfall in 2022 was concentrated in July bringing greater non-point source washout effects.

Figure 3B showed a significant correlation between monthly variations in TN concentrations and rainfall. During July and August, rainfall accounted for 60% of the annual total, and TN concentrations increased by 2.5 times compared to June. By September, rainfall decreased, and river runoff dropped rapidly by 74%. Despite the reduction in non-point source washout, point source discharge remained relatively stable, causing TN to maintain a relatively high concentration. Additionally, a portion of pollutants introduced during July and August may accumulate at the riverbed, forming sediment pollution. These newly deposited pollutants are more susceptible to resuspension caused by hydrodynamic forces, increasing the pollution load in the river (Hu et al., 2025; Wang et al., 2022). Runoff in October–November continued to decrease, however, the suitable temperature enhanced microbial degradation, promoting pollutant reduction. During December–February (the winter period), decreased microbial activity and degradation capacity, combined with the sedimentation of dead biomass, contribute to elevated TN concentrations in the river. From March to June, rainfall gradually increased; however, the capacity of runoff to mobilize pollutants remained lower than during the flood season, resulting in reduced pollutant input to the river. In addition, rising temperatures contributed to increased microbial activity and enhanced the self-purification capacity of the water body, promoting a decrease in TN concentrations.

Further, we performed high-frequency monitoring and analyzed daily variations in TN concentrations to examine the impact of rainfall (Figs. 3C and 3D). Clearly, TN concentrations increased rapidly following rainfall, confirming that rainfall is a key driver of TN variability. Particularly, when daily rainfall exceeded 100 mm, TN concentrations increased by a factor of 2 to 3.5 compared to pre-rainfall levels. Lower rainfall intensities led to smaller increases in TN concentrations, with delayed responses influenced by runoff generation processes. In contrast, high-magnitude rainfall events caused more pronounced elevations in TN concentrations. For example, during an extreme rainfall event of 278 mm/day, TN concentration increased by up to 14 times, exhibiting a real-time response between rainfall and water quality. As mentioned above, the flood season is a critical period for riverine TN variation and should receive increased attention in environmental management and control. Similarly, rainfall-driven TN increases have been observed in other Chinese rivers. Lin et al. (2024) documented 1–2 day lags under moderate to heavy rainfall. Dou et al. (2026) identified a baseflow threshold (0.92 mm) and shortened response time (0–1 days) under extreme rainfall. Wang et al. (2023a); Wang et al. (2023b); Wang et al. (2023c) reported that the highest pollution loads occurred in the abundant water period due to increased runoff and erosion. These patterns align with our findings, confirming the dominant role of flood-season rainfall in TN pollution. These consistencies suggest that rainfall-driven increases in TN concentration are a widespread phenomenon, reinforcing the need for flood-season management strategies beyond the Dai River alone. Therefore, rainfall drives the timing of TN pollution. Runoff, in turn, shapes its spatial distribution along the river. Next, we examined its spatial distribution. The spatial migration of nitrogen pollution in watersheds is driven by runoff from upstream to downstream.

### Spatial characterization and hotspot zones of riverine nitrogen pollution

**Figure 4:**
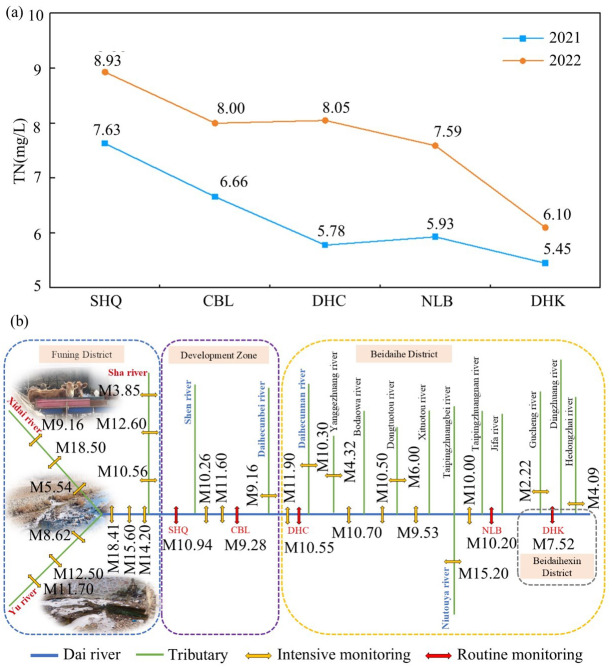
Spatial variations of TN concentration in the Dai river basin. (A) TN concentrations at the five routine monitoring sections (SHQ, CBL, DHC, NLB, DHK) in 2021 and 2022. (B) TN concentrations in the Dai river mainstream and all tributaries.

The spatial migration of nitrogen pollution in watersheds is driven by runoff from upstream to downstream. Monitoring stations located at river outlets are effective for assessing pollution at the watershed scale. However, accurately identifying pollution hotspots requires supplementary evidence. Unlike traditional analytical approaches, this study employed high-density monitoring data to detect pollution hotspots. First, the overall variation in TN concentration along the Dai river was examined. A 30% decrease in TN concentration was observed from the upstream SHQ section to the downstream DHK section (Fig. 4A). The high concentration of TN discharge in the upstream section was the primary factor contributing to the exceedance of TN concentration at the estuary.

Next, we analyzed the spatial distribution characteristics of nitrogen pollution and identified pollution hotspots using data from 51 intensive field sampling points along the Dai river. Figure 4B showed that TN concentrations in both the main river and its tributaries surpassed the Class V water quality standard, with the highest concentration exceeding the standard by 9.3 times and the lowest by 1.1 times. Overall, TN concentrations in the Dai river basin exhibited a decreasing trend from upstream to estuary. Upstream rivers (Yu river, Sha river, and Xidai river), midstream tributaries (Shen river, Daihecunbei river, and Daihecunnan river), and the downstream Niutouya river all demonstrated relatively high TN concentrations, ranging from 1.1 to 2.5 times those in the main Dai river and acting as primary sources of nitrogen pollution. To accurately identify pollution hotspots, we conducted field investigations to assess the surrounding environment of these rivers.

First, we analyzed the pollution conditions at the river headwaters. The TN concentration in the Yu river was 1.1 to 1.7 times higher than that at the DHK section. With a length of 12 km, it is one of the four major tributaries in the basin that exceed 10 km in length. Field surveys revealed that the riverbanks are characterized by villages, farmland, and orchards, with scattered livestock farming, which can easily release non-point source pollution during the flood season. In addition, domestic waste and sewage are discharged into the river, directly increasing the pollution load. The Xidai river exhibited TN concentrations 1.2 to 2.5 times higher than that of the DHK section, spanning 13 km and covering a large watershed area. The watershed contains multiple non-point pollution sources, including livestock farming, farmland, and vegetable cultivation, as well as the accumulation of trash in portions of the river segments. There is also untreated domestic sewage discharged in Yuguan town, contributing to river pollution. The Sha river has TN concentrations 1.4 to 1.78 times higher than those in the DHK section, extending 23 km, and is the largest tributary in the basin. Villages, farmland, and vegetable fields line the riverbanks of the Sha river, with rural domestic sewage being directly discharged into the water. Additionally, pollution from free-range poultry can be washed into the river during the flood season through runoff. Accumulated domestic waste is also found along the riverbank and within the river channel. Overall, the three headwater tributaries constituted critical pollution hotspots, accounting for approximately 48% of the Dai river watershed area and contributing significantly to the pollution load.

Next, we examined the pollution status in the middle and lower reaches of the Dai river. The TN concentration at the Shen river outlet was 1.2 times higher than that in the DHK section, and the river stretches over 14 km, contributing to the elevated TN load in the Dai river. Field investigations showed that pollution sources in the Shen river include domestic sewage, livestock farming, dry latrines, and fruit groves. Portions of the river channel were clogged with garbage, reducing the river’s self-purification capacity. In the midstream region, the Shen river constituted a pollution hotspot, contributing both higher TN concentrations and greater discharge volumes compared to other tributaries.

In summary, the headwater tributaries (Yu river, Xidai river, and Sha river), with high pollution concentrations and large watershed areas, were the primary hotspots affecting TN levels in the Dai river. The Shen river and Niutouya river in the middle and lower reaches followed as hotspots. These hotspot zones should be prioritized in future environmental management efforts. Following this, we further quantified the contributions of different nitrogen sources using the export coefficient model.

### Nitrogen pollution sources

**Figure 5:**
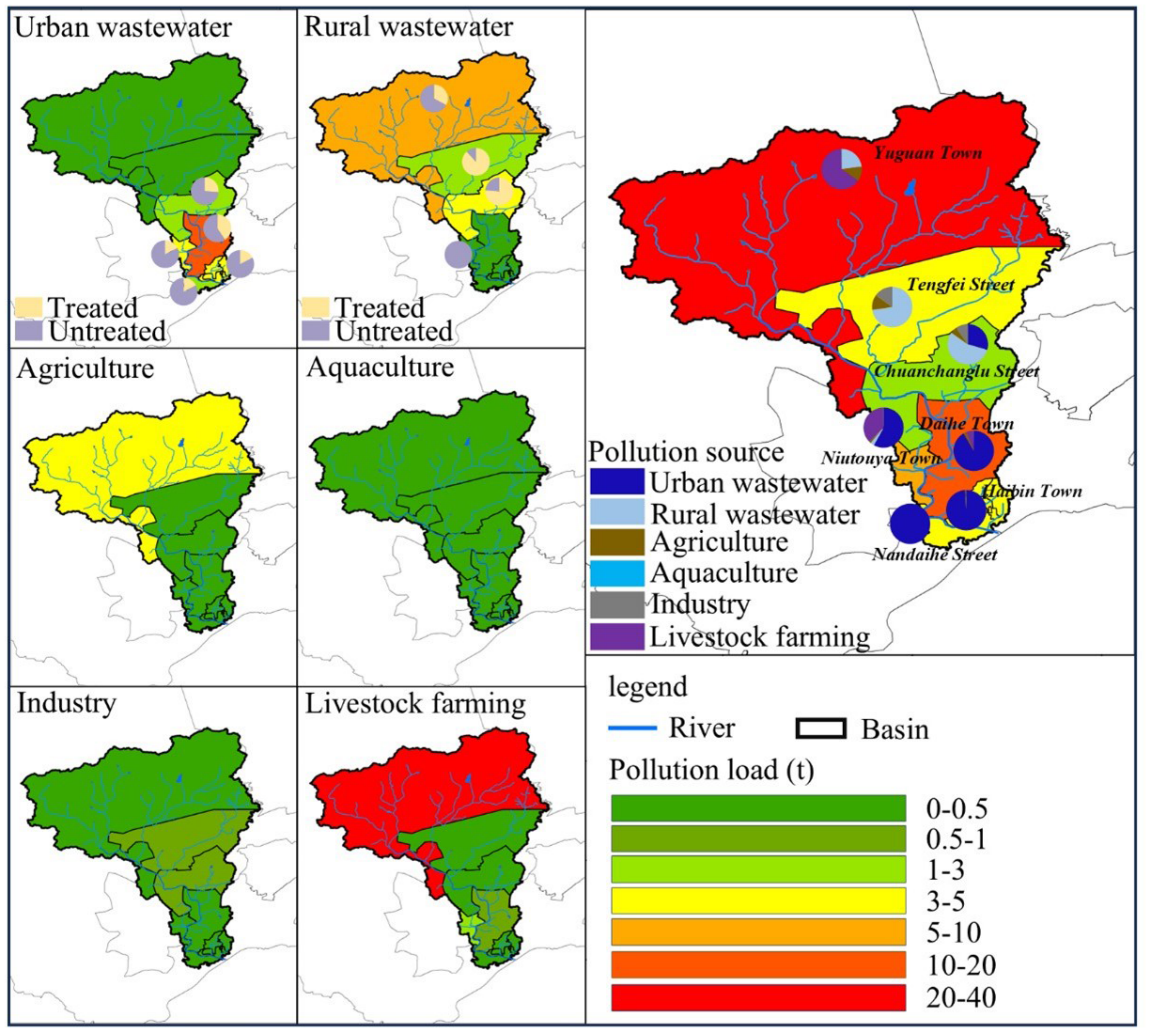
Spatial distribution of total nitrogen loads and source contributions across towns in the Dai river basin.

Land-based pollution input plays a significant role in river pollution. We employed the export coefficient method to assess the pollution sources in the Dai river. In China, environmental management is carried out by town-level governments. Most studies (Mao et al., 2023; Li et al., 2023a; Li et al., 2023b; Wang et al., 2024a; Wang et al., 2024b) analyzed land-based pollution inputs from urban, watershed, and provincial perspectives using statistical and remote sensing methods. In contrast to previous research, we focused on detailed investigations within the Dai river basin, narrowing pollution sources to the town level to facilitate more precise management. Subsequently, we assessed the sources of nitrogen pollution at the town level, including domestic sewage, agricultural non-point sources, livestock farming, aquaculture, and industrial discharges (Fig. 5).

 Domestic sewage discharge in towns includes both treated and untreated wastewater. Urban areas in the Dai river basin, mainly located in mid-downstream regions, achieve a 72% wastewater treatment rate, with treated wastewater discharges releasing 4.56 t of nitrogen pollution. However, untreated wastewater directly discharges 20.67 t of nitrogen pollution, demonstrating wastewater treatment plays a significant role in reducing nitrogen pollution. In regions with similar sewage treatment capacities, population size contributed to an increase in the volume of sewage discharge.

The urban population of Daihe town accounted for over 50% of the total population of the Dai river basin, and the nitrogen load from urban domestic wastewater contributed a comparable proportion. Notably, the discharge pathways of rural domestic wastewater differ significantly from those in urban areas. In rural regions, domestic wastewater is typically treated through decentralized treatment stations, which generally have low treatment rates and pollutant removal efficiency. Moreover, untreated wastewater is often discharged into rivers as non-point source pollution, in contrast to urban areas where wastewater is released through sewer networks as point source pollution. Wastewater treatment rates in towns across the Dai river basin ranged from 25% to 57%, significantly lower than those observed in urban areas. An exception was Niutouya town, which had higher treatment efficiency due to its connection to the municipal wastewater system. Calculations indicated that nitrogen discharges from treated rural domestic sewage in the Dai river basin were 10.28 t, while discharges from untreated rural domestic wastewater amounted to 3.67 t. The significantly higher nitrogen discharges from treated rural sewage compared to untreated wastewater are associated with relatively low pollutant removal capacity. Rural areas are mainly concentrated in the mid-upper reaches of the basin. Yuguan town contributed over 50% of the total rural nitrogen discharges in the Dai river basin. The highly urbanized downstream regions produced negligible rural domestic wastewater nitrogen discharge. Agricultural cultivation primarily consisted of cropland and garden land, covering an area of 4,964 ha, which accounted for 17.6% of the total area. Nitrogen pollution from agricultural activities amounted to 5.16 t, with Yuguan town contributing 75% of the total. Livestock farming, including pigs, sheep, and cattle, released 25 t of nitrogen pollution, with Yuguan town sharing 86.56% of the total discharge. The nitrogen pollution discharged from aquaculture and industrial enterprises was 0.18 tons and 0.6 tons, respectively, and relatively low.

Overall, urban domestic wastewater and livestock farming were the dominant sources of pollution in the Dai river basin, accounting for 35.87% and 35.55% of the total discharge, respectively. Wang et al. (2024a), Wang et al. (2024b) found that livestock and poultry breeding accounted for 24.08% of TN load in the Jialing River Basin. Xiao et al. (2021) reported that in the Tuojiang River basin, livestock and poultry breeding was the dominant source of TN non-point source pollution, with a contribution exceeding 45% during 2007–2017. Subsequent contributors included rural domestic wastewater (20.13%) and agricultural cultivation (7.34%). At the town level, Yuguan town discharged 33.34 t of nitrogen pollution, representing 47% of the total discharge. The primary pollution sources in Yuguan town were rural domestic wastewater and livestock farming. Both sources were difficult to control due to their widespread and scattered distribution and contributed to elevated pollution levels in the Yu river, Xidai river, and Sha river, consistent with the findings from intensive monitoring. The Monte Carlo uncertainty analysis showed that the TN load estimates for all source categories fell within the 95% confidence interval, which confirms the robustness of the source apportionment results. The analysis showed that urban wastewater and livestock dominated the TN load and should receive greater attention in management measures.

## Discussion

### Hotspot-informed management measures

**Figure 6:**
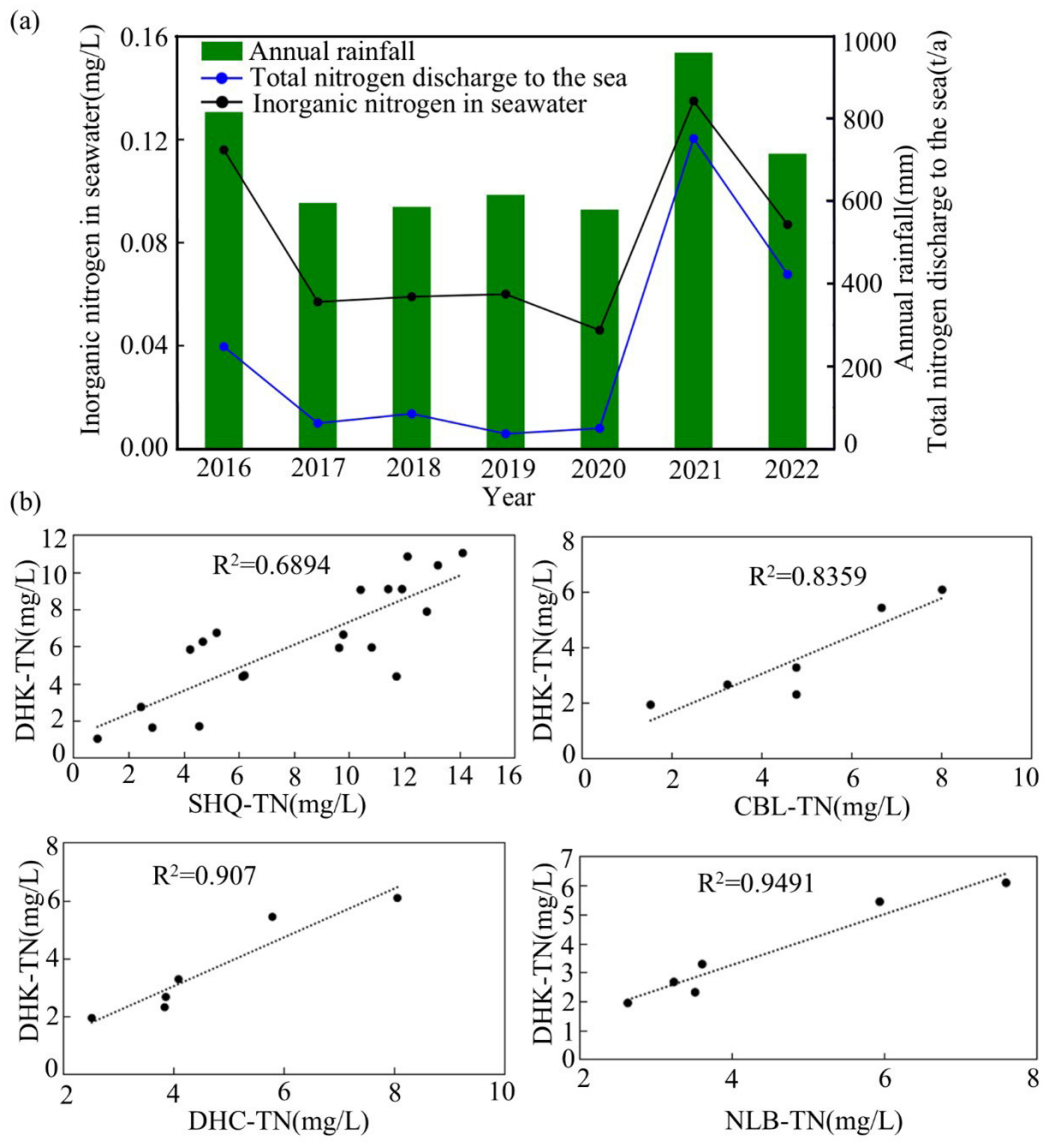
Relationships between hydrological forcing, riverine TN export, and coastal water quality, and the spatial correlation of TN along the Dai river. (A) Interannual trends in rainfall, riverine TN export, and coastal inorganic nitrogen (2016–2022); (B) Correlation of TN concentrations between the DKH estuary section and mainstream monitoring sections.

Land-based pollution migration is a process in which pollutants are carried by runoff from upstream areas to downstream regions. Land-based pollution export not only increased the nitrogen load in rivers but also impacted coastal marine environments (Fig. 6A; Wang et al., 2023a; Wang et al., 2023b; Wang et al., 2023c). Figure 6B showed a strong correlation between TN concentrations at the DKH section (estuary) and the main control sections of the Dai river, with this correlation becoming more pronounced in downstream areas. Additionally, the interannual variation trend of TN pollution in the Dai river aligned with changes in inorganic nitrogen concentrations in the nearshore seawater, confirming the impact of land-based pollution discharge on both river and seawater environments. Land-based pollution involves diverse contaminant types and complex environmental impacts. To effectively reduce pollution, we further proposed management and control strategies based on hotspot diagnosis.

This study revealed that the flood season was a critical period for riverine TN pollution, that Yuguan town was a pollution hotspot, and that urban domestic wastewater and livestock farming were the dominant sources. Following the hotspot analysis, we explored differentiated management and control strategies for each town. The primary pollution sources in Yuguan town (headwater) were rural domestic wastewater and livestock farming. As a result, the three major tributaries became polluted. The rural domestic wastewater treatment rate in Yuguan town at the river headwaters reached 56.9%, while the pollution removal rate was only 47%. In contrast, the urban domestic wastewater treatment rate in the same town achieved 72%, with the pollution removal rate exceeding 70%. Considering the decentralized nature of rural domestic wastewater discharge in Yuguan town, the construction of wastewater pipe networks should be improved to enhance treatment rates. Additionally, secondary treatment of tailwater and water reuse systems could be strengthened to achieve both resource utilization and pollution reduction. Furthermore, accumulated garbage in side ditches, pipes, and ponds should be cleared before rainfall, and dry toilets near the river should be centrally cleaned to avoid the direct discharge of pollution into the river during rainfall. For livestock farming pollution control, it is recommended that large-scale breeding farms implement solid–liquid separation of manure waste, and improve manure treatment facilities. Meanwhile, resource utilization models for manure treatment should be explored, such as returning manure to fields, utilizing sewage as fertilizer, producing biogas, and generating power (Lu, Ma & Shao, 2024). Agricultural pollution reduction can be integrated with livestock and poultry manure treatment to create a circular model of ‘planting–breeding–organic fertilizer production–agricultural planting.’ Furthermore, fertilization based on soil testing and efficient irrigation practices should be promoted to reduce pollution discharge. Tengfei street (town level), located in the middle reaches of the river, mainly faced challenges related to rural domestic wastewater. Similar measures can be adopted with reference to those proposed for Yuguan town. In addition, Tengfei street (town level) also experienced problems related to industrial enterprise wastewater, for which it is recommended to strengthen automatic monitoring capacity and strictly control unauthorized pollutant discharge. In Chuanchanglu street (town level), rural and urban domestic wastewater were the dominant sources, contributing 32.8% and 61.2% of the TN discharge, respectively. The management and control strategies for rural domestic wastewater were explored in Yuguan town. For Chuanchanglu street, the focus was on controlling urban domestic wastewater. Unlike rural domestic wastewater, urban domestic wastewater discharges into the river as a point source and may become the primary factor in river pollution during non-flood seasons when river runoff is relatively low. Therefore, urban domestic wastewater management needs to complete the wastewater pipeline network to ensure that all domestic wastewater is effectively collected and treated at wastewater treatment plants. Additionally, wastewater treatment plant discharge standards should be explored based on river management goals and the water environmental capacity, to protect the river water environment. Haibin town, Daihe town, and Niutouya town are located in the downstream section of the river and face challenges related to urban domestic wastewater. To address this issue, it is necessary to complete the construction of sewer networks and optimize wastewater treatment standards. Additionally, these towns are affected by livestock farming. Given their urbanization characteristics, it is recommended to use economic incentives to reduce individual farming practices and promote the establishment of large-scale farms with centralized wastewater treatment systems.

The flood season is the main driver of TN pollution in the Dai river basin. Pre-rain removal of waste and enhanced storm-time treatment can reduce flood-season nitrogen loads. The upstream hotspots (Yu, Xidai, and Sha rivers) are dominated by urban wastewater and livestock farming, calling for rural sewer networks, manure separation, and routine farm inspections. The strong river-coast nitrogen link implies that reducing land-based nitrogen directly benefits coastal ecosystems. Thus, an integrated upstream-downstream strategy is needed. Our town-scale source apportionment provides a spatially explicit basis for differentiated policies and can be extended to other data-limited small rivers.

## Conclusions

This study proposed a framework for identifying spatiotemporal pollution hotspots and dominant sources in the Dai river by integrating intensive monitoring with the export coefficient method. TN pollution in the Dai river remained severe, with multi-year average concentrations exceeding the Class V water quality standard (GB3838-2002). In terms of temporal variation, data from routine monitoring stations revealed that the flood season was a critical period for increases in riverine TN concentrations. Inter-annual variations in TN, influenced by rainfall, exhibited a trend of initial decline followed by a subsequent increase. Additionally, a lag effect was observed in monthly-scale fluctuations of TN. Rainfall events had a pronounced impact on water quality, with TN concentrations increasing by up to 14 times compared to pre-rainfall levels. Regarding spatial variation, TN concentrations in the Dai river decreased by approximately 30% from the upstream SHQ section to the downstream DHK section. Intensive monitoring revealed that TN concentrations in the Yu river, Xidai river, and Sha river—located at the headwaters—were 1.1 to 2.5 times higher than those observed at the Dai river estuary. Additionally, these tributaries, characterized by large watershed control areas, were identified as key hotspots contributing to nitrogen pollution in the Dai river. The export coefficient model results showed that urban domestic wastewater and livestock were the primary sources of pollution, accounting for 35.87% and 35.55% of total discharge, respectively. Rural domestic wastewater was the following contributor, accounting for 20.13% of total discharge, while agricultural sources accounted for a smaller share of only 7.34%. Specific to the towns, Yuguan town discharged 33.34 t of nitrogen pollution, accounting for 47% of the total discharge. The results from the export coefficient model were consistent with those obtained from intensive monitoring. Further, we proposed integrated management measures tailored to the various pollution sources in each town. The case study of Daihe demonstrates that our method effectively identifies sensitive temporal and spatial hotspots of river pollutants, as well as the major pollution sources.

## Supplemental Information

10.7717/peerj.21512/supp-1 Supplemental Information 1 Raw data of annual, monthly, and daily trends of TN pollution and hydrological variations in the Dai river

10.7717/peerj.21512/supp-2 Supplemental Information 2 Raw data of spatial variations of TN in the Dai river

10.7717/peerj.21512/supp-3 Supplemental Information 3 Raw data of sources of TN pollution in the Dai river

10.7717/peerj.21512/supp-4 Supplemental Information 4 Raw data of correlations of TN pollution in the Dai river

## Additional Information and Declarations

### Data Availability

The following information was supplied regarding data availability: The raw data is available in the Supplemental Files.
